# The Influence of Cold Forming and Heat Treatment Processes on the Mechanical and Fracture Properties of AA6016 Aluminum Sheets

**DOI:** 10.3390/ma17092074

**Published:** 2024-04-28

**Authors:** Baitong Liu, Jiahong Lu, Shiyao Huang, Zuguo Bao, Xilin Li, Zhenfei Zhan, Qing Liu

**Affiliations:** 1 College of Materials Science and Engineering, Nanjing Tech University, Nanjing 211816, China; 202161203294@njtech.edu.cn (B.L.); 202161203189@njtech.edu.cn (J.L.); baozuguo@njtech.edu.cn (Z.B.); 2 Yangtze Delta Advanced Materials Research Institute, Suzhou 215133, China; m13550509099@163.com; 3 College of Mechanical and Vehicle Engineering, Chongqing Jiaotong University, Chongqing 400074, China; zhenfei_zhan@163.com

**Keywords:** AA6016, pre-strain, heat treatment, precipitation hardening, backpropagation (BP) neural network

## Abstract

In order to ascertain the mechanical properties and fracture performance of AA6016 aluminum sheets after cold forming and heat treatment processes, uniaxial tensile tests and fracture tests were conducted under various pre-strain conditions and heat treatment parameters. The experimental outcomes demonstrated that pre-strain and heat treatment had significant impacts on both stress–strain curves and fracture properties. Pre-strain plays a predominant role in influencing the mechanical and fracture properties. The behavior of precipitation hardening under different pre-strains was investigated using Differential Scanning Calorimetry (DSC). The results indicated that pre-strain accelerates the precipitation of the β″ strengthening phase, but excessive pre-strain can inhibit the heat treatment strengthening effect. To consider the influences of pre-strain and heat treatment, a constitutive model, as well as a predictive model for load–displacement curves, was established using a backpropagation (BP) neural network. An analysis of the number of hidden layers and neuron nodes in the network revealed that the accuracy of the model does not necessarily improve with an increase in the number of hidden layers and neuron nodes, and an excessive number might actually decrease the efficiency of the machine learning process.

## 1. Introduction

As the global energy crisis intensifies, the automotive industry is shifting towards the development of more environmentally friendly, safe, and energy-efficient vehicles. In this context, the use of lightweight materials has become an effective method to achieve vehicle lightweighting. Aluminum alloys, as a key lightweight material, are widely used in automobile bodies. Typically, the body manufacturing process involves stamping and painting processes, which can alter the mechanical and fracture properties of the sheet material.

Pre-treatment has a significant influence on the mechanical properties of aluminum alloys. From a macroscopic perspective, pre-strain and heat treatment result in an increase in the strength of aluminum sheets, accompanied by a reduction in plasticity. Additionally, these treatments tend to lower the anisotropy of the material after pre-strain and heat treatment [1,2]. On a microscopic level, pre-strain and pre-aging promote the rapid formation of solute clusters, but inhibit their growth during prolonged aging. The precipitated β″ strengthening phase mitigates natural aging, thus enhancing the heat treatment effect [3,4,5]. Nevertheless, the impact of pre-strain and heat treatment on the fracture performance of aluminum alloys is less frequently reported, and research in this field is still not exhaustive.

Constitutive models have been widely used in the study of aluminum alloy formation due to its ability to reflect the relationship between stress and strain during material deformation. Models that simultaneously consider pre-strain and heat treatment include the H-S flow stress model [2,6] and the combination of the YLD2000-2d yield criterion with the Swift–Voce hardening model [7]. These models were calibrated by fitting stress–strain curves obtained under different pre-strain and heat treatment conditions. Due to numerous model parameters, the fitting process is quite complex, and such constitutive models cannot consider pre-strain and heat treatment parameters as independent variables, limiting their extrapolative predictive abilities. Conversely, artificial neural network models, particularly backpropagation (BP) neural networks, are increasingly favored for their efficiency in considering multiple factors [8,9,10,11].

Fracture models, known for effectively describing material fracture behavior under various stress states, are extensively utilized in researching aluminum alloy fracture behavior. Notably, there has been research on the Hosford–Coulomb failure model after pre-strain and heat treatment [7]. By calibrating the parameters of the Hosford–Coulomb failure model using the experimentally obtained force–displacement curves, the pre-strain- and post-heat-treatment Hosford–Coulomb failure model can be obtained. In this methodology, the force–displacement curves are particularly crucial. Generating these curves necessitates extensive fracture testing post pre-strain and heat treatment, which requires considerable time and human resources. However, with the aid of artificial neural network models, it is possible to develop predictive models for force–displacement curves after pre-strain and heat treatment.

Most of the reports related to constitutive models have focused on fitting test results, and process parameters (e.g., pre-strain, heat treatment time and heat treatment temperature) were not used as input variables. Therefore, it is impossible to predict the stress–strain curves under different process parameters. On the other hand, force–displacement curves were mostly obtained by testing. The prediction of force–displacement curves under different process parameters has received limited attention. This study will examine the impact of pre-strain, heat treatment temperature, and heat treatment time on the mechanical and fracture properties of AA6016 sheet materials via uniaxial tensile tests and shear fracture tests. Subsequently, this paper aims to develop a constitutive model incorporating the influences of pre-strain and heat treatment, along with predictive models for force–displacement curves of various fracture specimens, employing a BP neural network. The research will also explore how the configuration of neural network model parameters affects the accuracy and efficiency of the fit. Additionally, this research will examine the impact of genetic algorithms on the accuracy of model predictions.

## 2. Experimental Section

### 2.1. Experimental Design

This paper utilizes AA6016 aluminum sheets with a thickness of 1 mm. The composition of the material was provided by Southwest Aluminium, Chongqing, China, as shown in Table 1. An orthogonal experimental design was adopted for determining pre-strain and heat treatment parameters, and is outlined in Table 2.

### 2.2. Uniaxial Tensile Test

**Figure 1:**
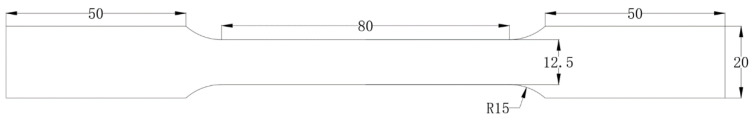
Uniaxial tensile specimen.

In this section, the mechanical properties of uniaxial tensile specimens made from the original sheet material were examined. Subsequently, the sheet material was pre-stretched at various pre-strain levels. Following pre-stretching, the specimens were subjected to heat treatment according to the orthogonal test table (as shown in Table 2). Finally, the specimens were pulled to fracture. The test was repeated three times for each condition. The uniaxial tensile tests were conducted on a 5982 universal testing machine manufactured by Instron, Boston, MA, USA. The dimensions and shape of the specimens are depicted in Figure 1.

### 2.3. Fracture Test

**Figure 2:**
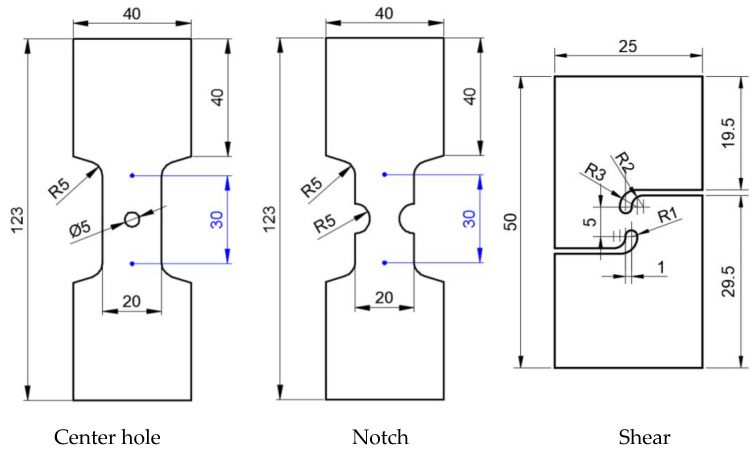
Fractured specimen.

In this section, pre-stretching experiments were first conducted on strip plates of two sizes: 210 mm × 50 mm and 130 mm × 35 mm. After pre-stretching, the sheets were wire-cut to the dimensions depicted in Figure 2. The specimens prepared were heat-treated following the process specified in Table 2 and subsequently stretched to fracture. These fracture tests were conducted on an Instron 5982 universal testing machine. Furthermore, to obtain more precise displacement data, Digital Image Correlation (DIC) was installed in this section to record displacement and strain fields. DIC is a non-contact method and remains accurate in recording the changes in displacement and strain fields, especially during unstable deformations like necking in the specimens.

### 2.4. DSC Experiments under Different Pretreatment Conditions

To determine the precipitation behavior of the AA6016 aluminum alloy under different pre-treatment conditions, this section included the preparation of the pre-treated samples into circular discs with a diameter of 3 mm, followed by Differential Scanning Calorimetry (DSC) testing on a DSC214 manufactured by Netzsch, Selb, Germany. This method involves operating the 1100LF system under an argon atmosphere, with a temperature range from 50 to 550 °C and a heating rate set at 10 °C/min. Such an approach helps researchers to understand the thermal properties and precipitation behavior of the material.

## 3. BP Neural Network Model

**Figure 3:**
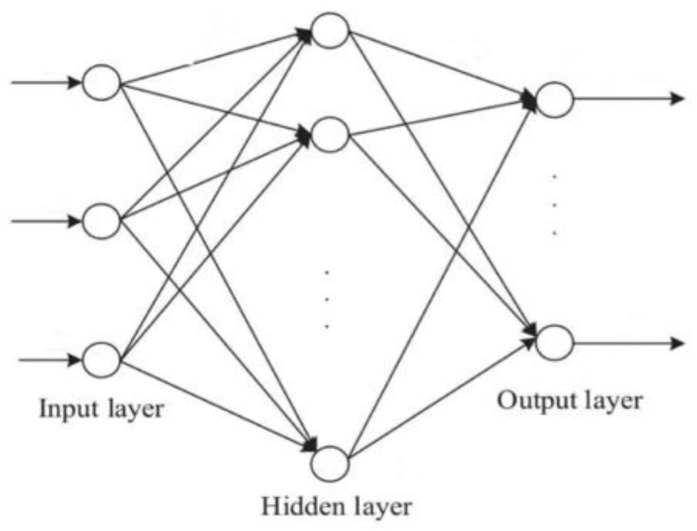
BP neural network structure.

**Figure 4:**
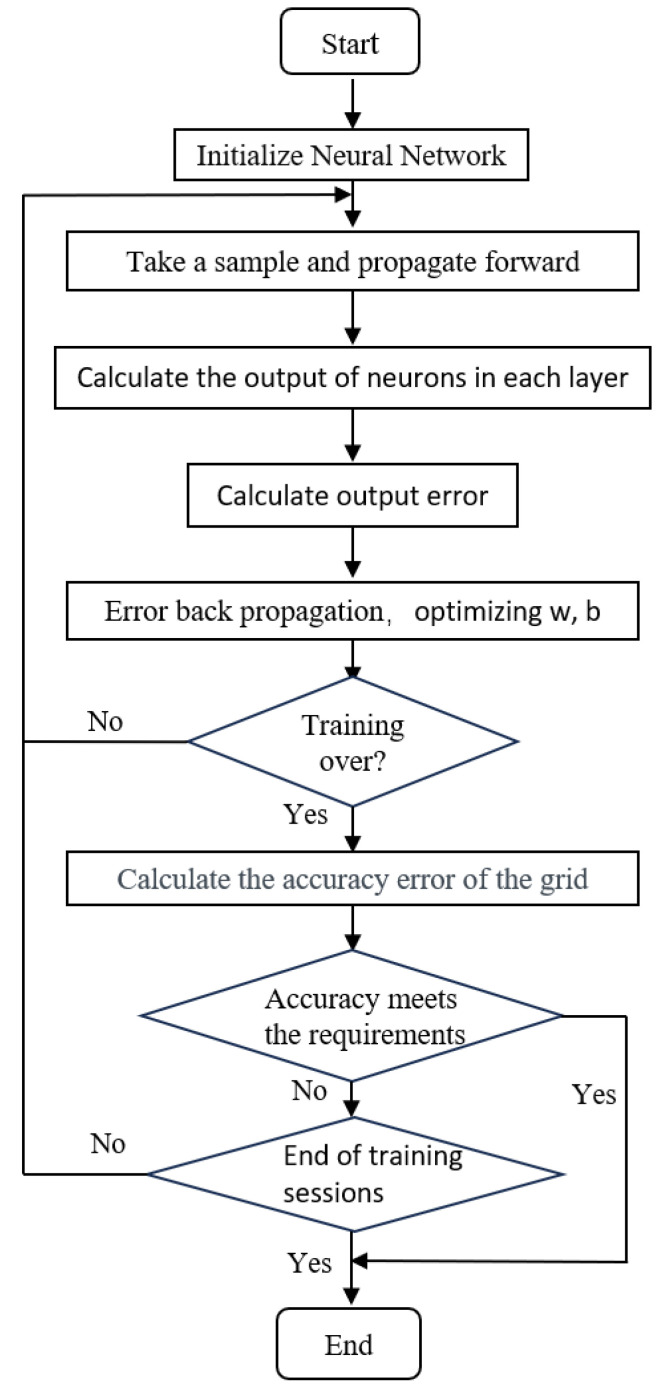
Neural network flowchart.

The BP neural network is a common neural network model used for solving classification and regression problems. The BP network can be employed to learn and store the mapping relationship of a large number of input–output models without the need to explicitly describe these mapping relationships using mathematical equations [12,13]. This model mainly consists of an input layer, an output layer, and hidden layers, as shown in Figure 3, where the inputs are connected to the outputs through neurons. The training process of the BP neural network consists of forward propagation and error backpropagation. During forward propagation, the input layer inputs the samples, and the hidden layer computes the output. When the output does not match the actual result, it leads to the generation of the mean square error, initiating the backward propagation phase. During backpropagation, the output error is calculated and propagated backwards through the hidden layers to the input layer, distributing the error among all units. The error signals of each layer’s units serve as the basis for adjusting the unit weights. The weights and bias parameters are then learned using gradient descent. The entire model flowchart of the neural network is shown in Figure 4. The specific processing steps of the model will be elaborated upon in the following text.

### 3.1. Pretreatment

First, to establish the model, it is necessary to normalize the raw data, mapping it to the range of [−1, 1] using Formula (1) [14]. This model is built in Python, and the data normalization is achieved using the MinMaxScaler function in the library.

(1) $$X^{\prime }=\frac{2*\left(X-X_{min}\right)}{\left(X_{max}-X_{min}\right)}-1$$

In Formula (1), $X^{\prime }$ represents the normalized original data, $X_{max}$ represents the maximum value in the sample data, $X_{min}$ represents the minimum value in the sample data, and X represents the sample data.

### 3.2. Selection of Parameters

The number of input layer neural nodes, denoted as m, and the number of output layer neural nodes, denoted as n, are determined by the respective quantities of input and output in the dataset. The number of hidden layers and the neural node count in each layer are determined through trial and error, taking into account the training quality and efficiency of the model. The hidden layers can consist of a single hidden layer or multiple hidden layers, with the range in this paper is between 1 and 3. The range for the number of neural nodes in the hidden layer is between 5 and 20.

### 3.3. Selection of Activation Function

As part of the neural network, the activation function is responsible for introducing non-linearity to the neurons. The Sigmoid function is widely used because its activation logic is closest to that of neurons. Therefore, in this model, the Sigmoid function [13] is chosen as the activation function, and its functional formula is shown as (2). The derivative formula of the Sigmoid function is shown as (3).

(2) $$f\left(x\right)=\frac{1}{1+e^{-x}}$$

(3) $$f^{\prime }\left(x\right)=f(x)(1-f\left(x\right))$$

Input data x in the array may contain large negative absolute values, and passing this to the Sigmoid function will result in a very large value for $e^{-x}$ in the denominator, causing $e^{-x}$ to overflow. That is, when the input data x<0, the function formula is as shown in (4).

(4) $$f\left(x\right)=\frac{e^{x}}{1+e^{x}}$$

### 3.4. Model Evaluation Indicators

*R*^2^ is a statistical metric that evaluates the goodness of fit of the predictive data. When *R*^2^ is closer to 1, it indicates a better predictive capability of the model. Its calculation formula is shown in Equation (5) [9]. Root Mean Square Error (RMSE) is a statistical metric used to measure the prediction error of a model. It represents the root mean square of the errors between predicted values and actual observed values, and its calculation formula is shown in Equation (6) [9].

(5) $$R^{2}=1-\frac{SSR}{SST}=1-\frac{\sum_{i=1}^{n}{\left(T_{i}-P_{i}\right)}^{2}}{\sum_{i=1}^{n}{\left(T_{i}-\bar{T_{i}}\right)}^{2}}$$

(6) $$RMSE=\sqrt{\frac{\sum_{i=1}^{n}{\left(P_{i}-T_{i}\right)}^{2}}{n}}$$

where SSR is the sum of squares due to regression, SST is the total sum of squares, $T_{i}$ is the observed value of the dependent variable, $P_{i}$ is the predicted value of the dependent variable from the model, $\bar{T_{i}}$ is the mean of the dependent variable, and n is the sample size.

## 4. Results and Discussion

### 4.1. Experimental Results Analysis

#### 4.1.1. Uniaxial Tensile Test Results and Analysis

**Figure 5:**
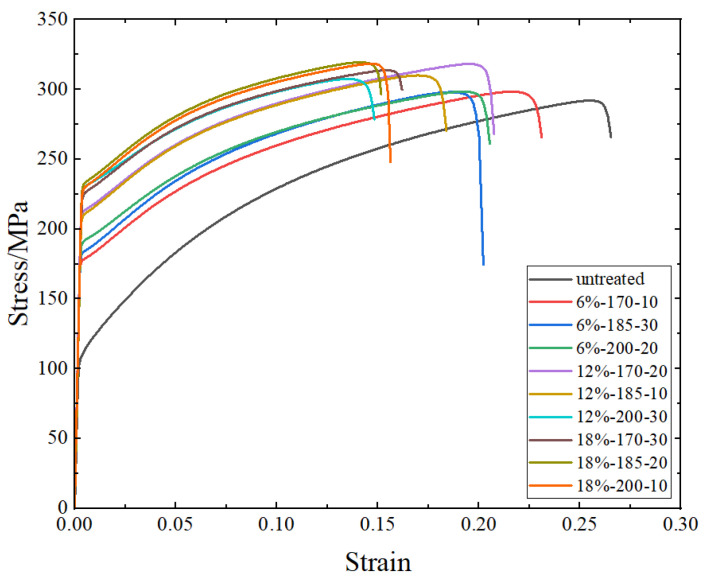
AA6016 true stress–strain curves before and after pre-strain and heat treatment.

Figure 5 shows the stress–strain curves of AA6016 before and after pre-strain and heat treatment. From the stress–strain curves, it can be observed that pre-strain and heat treatment have a significant impact on the yield strength of AA6016. Both yield strength and tensile strength increased after pre-strain and heat treatment, while the elongation at break decreased.

Table 3 shows the yield strength, tensile strength, and elongation at break of the initial sheet metal, while Table 4 presents the yield strength, tensile strength, and elongation at break of AA6016 after different pre-strain and heat treatments (the standard deviation formula is shown in Formula (7)). Compared to the untreated sheet metal, the sample with a pre-strain level of 18% and a heat treatment process at 185 °C for 20 min showed the highest increase in yield strength and tensile strength, with a 124.4 MPa increase in yield strength and a 48.1 MPa increase in tensile strength. As strength increases, plasticity decreases, resulting in a 16% decrease in elongation at break. From Table 4, it can be observed that at pre-strain levels of 6% and 12%, with an increase in baking temperature and baking time, the strength of the material improves. However, at a pre-strain level of 18%, this trend is not observed. This phenomenon will be explained in Section 4.1.3.

(7) $$S=\sqrt{\frac{\sum_{i=1}^{n}{\left(x_{i}-\bar{x}\right)}^{2}}{n-1}}$$

where n represents the amount of data and $\bar{x}$ represents the average value of the data.

**Figure 6:**
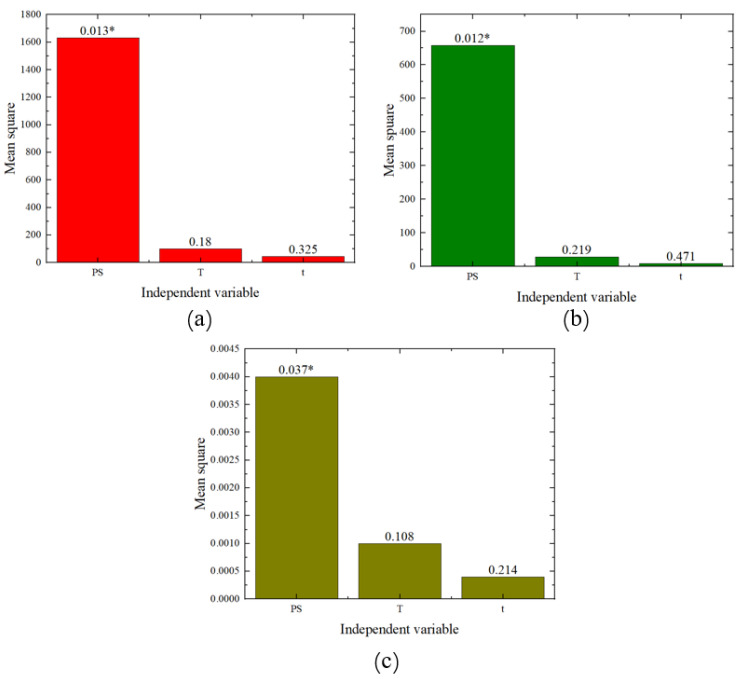
Variance analysis chart: (**a**) yield strength, (**b**) tensile strength, (**c**) elongation.

In order to determine the extent and priority of the impact of pre-strain, heat treatment temperature, and heat treatment time on the mechanical properties of AA6016, this study conducted variance analysis and range analysis on the experimental results. Analysis of variance is a statistical method used to analyze the effect of different factors on the variation of data. When the *p*-value is less than 0.05, it indicates that the factor has a significant effect on the results of the test. Range analysis is also a statistical method for analyzing the effects of different test factors on test results in orthogonal tests. When the difference between the maximum and minimum values of the data is larger, it indicates that the factor has a greater influence on the results of the test. The results of the analysis of variance are shown in Figure 6 (where PS represents pre-strain, T represents heat treatment temperature, and t represents heat treatment time). From the results, it can be observed that the *p*-values (the numbers on the bar charts, with * indicating a *p*-value of less than 0.05) for the pre-strain samples in the analysis of yield strength, tensile strength, and elongation are all less than 0.05, indicating significant differences for pre-strain in these properties. On the other hand, the *p*-values for the analysis of heat treatment time and temperature in relation to yield strength, tensile strength, and elongation are all greater than 0.05, suggesting that heat treatment time and temperature do not exhibit significant differences in these properties. Therefore, from the charts, it can be seen that the primary and secondary orders of influence on yield strength, tensile strength, and elongation are pre-strain, heat treatment temperature, and heat treatment time.

**Figure 7:**
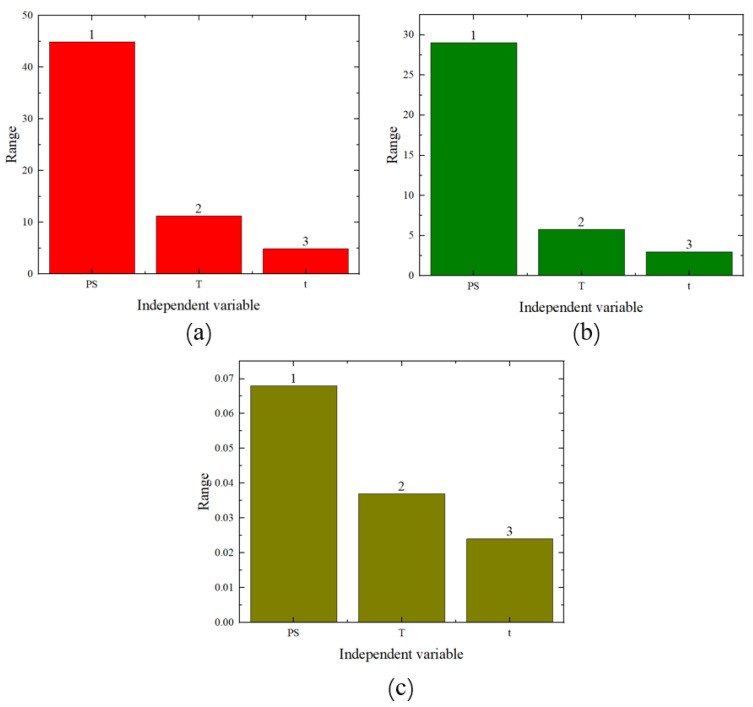
Range analysis chart: (**a**) yield strength, (**b**) tensile strength, (**c**) elongation.

The range analysis results are shown in Figure 7 (where the numbers on the bar charts represent the ranking). From these figures, it can be seen that among the three factors, the primary influencing factor is pre-strain, followed by heat treatment temperature and heat treatment time. Therefore, it is evident that both the analysis of variance and the range analysis results demonstrate that pre-strain has the greatest impact on the mechanical performance.

#### 4.1.2. Fracture Test Results and Analysis

**Figure 8:**
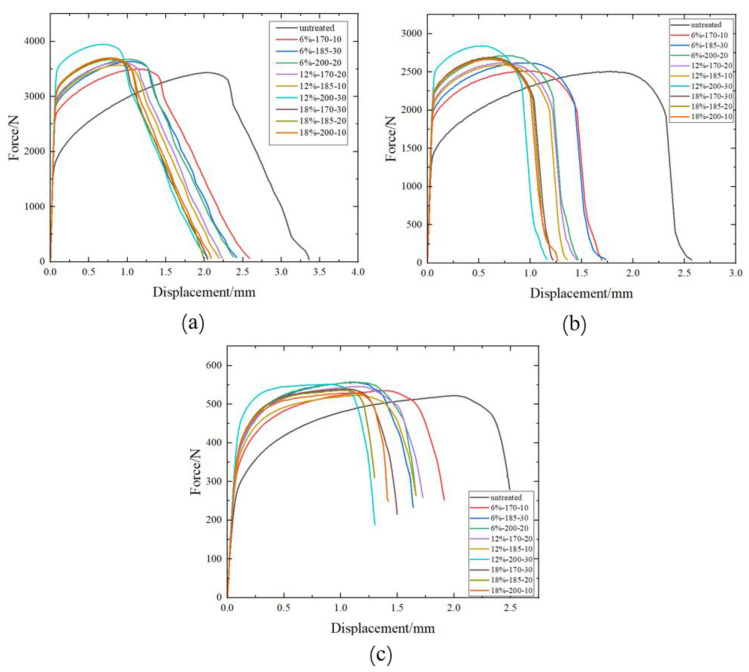
The force–displacement curves of three fracture specimens before and after pre-strain and heat treatment. (**a**) Center hole specimen, (**b**) notch specimen, (**c**) pure shear specimen.

Figure 8 shows the force–displacement curves of three fracture specimens before and after pre-straining and heat treatment. From the graphs, it is evident that for AA6016, after pre-strain and heat treatment, the maximum load increases, while the failure displacement decreases. It is also noticeable that all three fracture specimens achieve their maximum load and the greatest reduction in failure displacement at a pre-strain level of 12% and a heat treatment temperature of 200 °C. The center hole specimen, at a pre-strain level of 12% and a heat treatment process of 200 °C for 30 min, experienced an increase in maximum load by 514.1 N, while the failure displacement decreased by 2.0 mm. For the notched specimen, under the same pre-strain level and heat treatment, the maximum load increased by 332.7 N, and the failure displacement decreased by 1.4 mm. Finally, for the pure shear specimen, at the same conditions, the maximum load increased by 29.3 N, and the failure displacement decreased by 1.2 mm. Similar to Section 4.1.1, at pre-strain levels of 6% and 12%, an increase in baking temperature and baking time lead to an enhancement in the maximum load required for the fracture of the test specimens. However, this trend was not observed at the 18% pre-strain level. This phenomenon will also be explained in Section 4.1.3.

**Figure 9:**
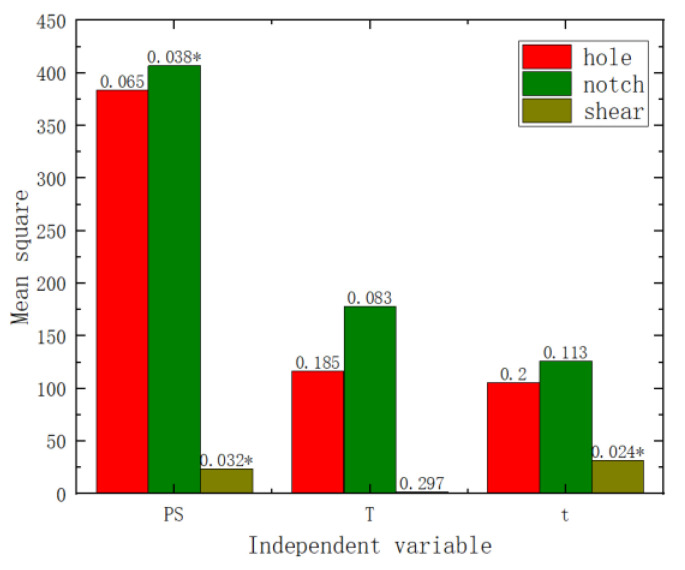
Shear strength variance analysis chart.

**Figure 10:**
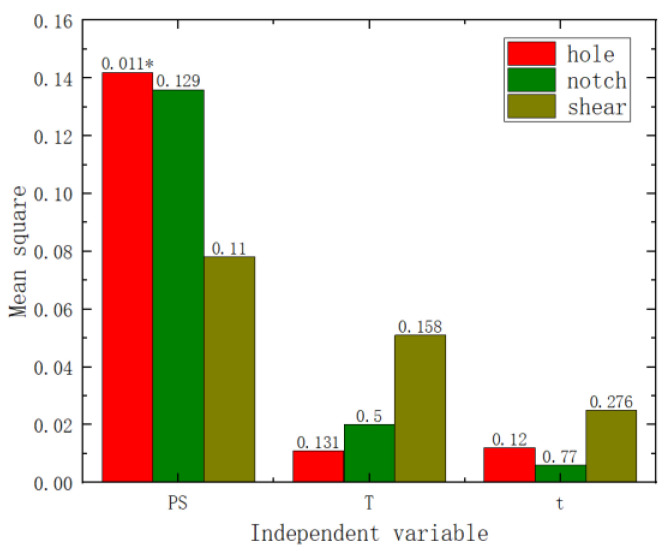
Failure displacement variance analysis chart.

**Figure 11:**
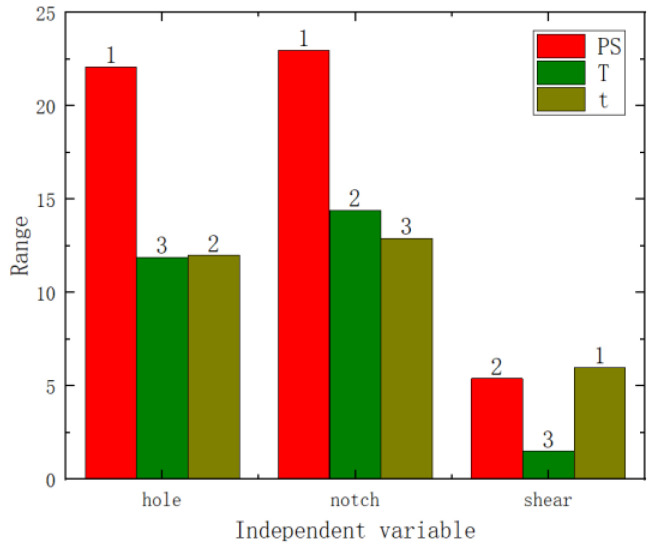
Shear strength range analysis chart.

**Figure 12:**
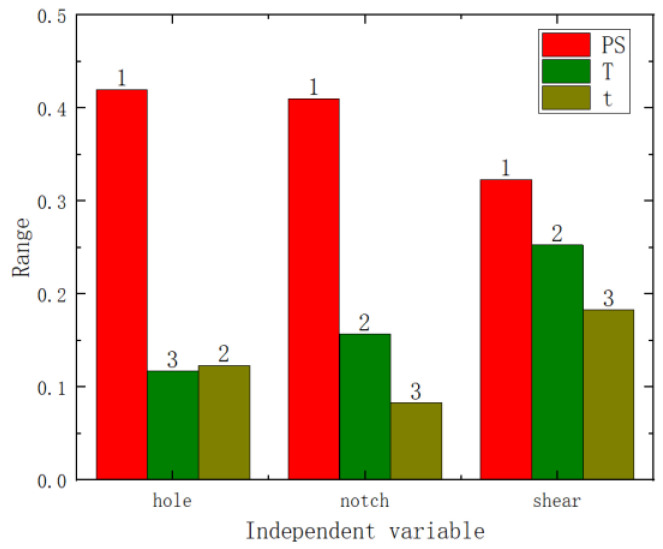
Failure displacement range analysis chart.

Variance and range analyses were conducted on the failure displacement and shear strength of the three specimen types with respect to pre-strain and heat treatment parameters. From the *p*-values in Figure 9 and Figure 10 (the numbers on the bar charts, with * indicating a *p*-value of less than 0.05), it can be observed that pre-strain significantly affected the failure displacement of the center hole specimen, the notched specimen, and the pure shear specimen. Heat treatment temperature did not exhibit a significant effect on the three types of fracture specimens. However, the heat treatment time showed a significant impact on the shear strength of the pure shear specimen. Range analyses results are shown in Figure 11 and Figure 12 (where the numbers on the bar charts represent the ranking). It can be observed that pre-strain significantly affects the shear strength and failure displacement of the three types of specimens. The influence of heat treatment temperature and time varies slightly for different specimens. In particular, for the pure shear specimen, its shear strength is notably influenced by heat treatment time, to the extent of slightly surpassing the impact of pre-strain. It can be seen that similar to the results of uniaxial tension, pre-strain predominantly influences the fracture performance of AA6016.

#### 4.1.3. DSC Test Results Analysis

**Figure 13:**
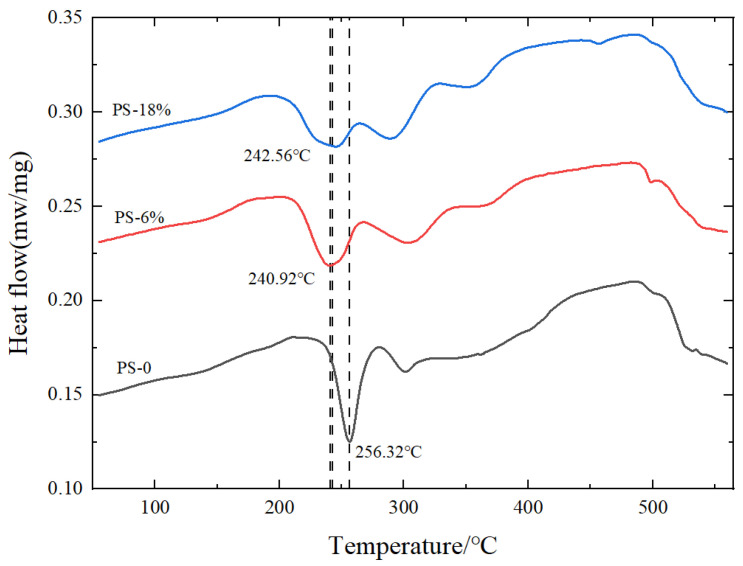
DSC curves under different pretreatment conditions.

Figure 13 shows the DSC curves under different pretreatment conditions. The endothermic and exothermic phenomena in the figure represent precipitation at different temperatures. It can be seen from the figure that the sample without pre-strain has a dissolution trough of solute clusters between 180 °C and 220 °C, and an endothermic peak forms around 240 °C, which is related to the formation of the GPI region (β′). At around 256 °C, an exothermic peak forms, which is related to the formation of the GPII region (β″) [5,15,16].

Compared to samples without pre-strain, the dissolution trough between 180 °C and 220 °C in pre-strained samples is significantly reduced or eliminated. This is because pre-strain can effectively inhibit natural aging. With an increase in pre-strain level, the formation temperature of the exothermic peak (β″ strengthening phase) decreases. This is due to the increase in dislocation density in pre-strained samples, where these high-energy dislocation points accelerate the movement of quenched vacancies to the GPI region [17,18], making it more favorable for the formation of the β″strengthening phase, thereby enhancing the strength of pre-strained samples. From Figure 13, it can be observed that the precipitation temperature of the exothermic peak for both 6% and 18% pre-strain levels is lower than the precipitation temperature of the endothermic peak in samples without pre-strain. Under 0–6% pre-strain, the precipitation temperature of the exothermic peak shows a decreasing trend. At an 18% pre-strain level, the precipitation temperature of the exothermic peak is slightly higher than that at 6%. This is because at 18% pre-strain, the material has a higher dislocation density. When the dislocation density is high, dislocation entanglement occurs, leading to uneven distribution of precipitated phases [5,19], inhibiting the heat treatment strengthening effect, limiting the improvement in strength.

Based on classical nucleation theory, the nucleation rate can be obtained from Equation (8) [20,21].

(8) $$J=N_{0}Z\upsilon exp(-\frac{∆G^{*}}{K_{B}T})\eta (t)$$

where $N_{0}$ is the number of nucleation sites, Z is the Zeldovich factor, υ is the atomic attachment rate, $∆G^{*}$ is the nucleation free activation enthalpy, $K_{B}$ is the Boltzmann constant, T is the absolute temperature, and η(t) is the activation factor. Assuming that all independent variables except the number of nucleation sites remain constant, an increase in dislocation density leads to an increase in the number of nucleation sites and, consequently, the nucleation rate, ultimately resulting in greater precipitation of the β″ strengthening phase.

If only considering the volume free energy and surface energy, the Gibbs free energy of the nucleation process can be obtained from Equation (9) [20].

(9) $$∆G^{*}=∆g_{T}\frac{4\pi }{3}r^{3}+4\pi r^{2}\gamma$$

In this equation, $∆g_{T}$ represents the volume free energy, γ is the surface energy, and r is the radius of the precipitated phase. When dislocations occur, this process, due to its uneven occurrence, leads to a decrease in the nucleation free energy $∆G^{*}$, resulting in faster nucleation [22]. Assuming constant volume free energy and surface energy, the decrease in nucleation free energy $∆G^{*}$ will lead to a decrease in the radius of the precipitated phase, thereby resulting in more precipitation of the β″ strengthening phase.

The above analysis shows that the introduction of appropriate pre-strain will increase the precipitation rate and quantity of the β″ strengthening phase, thereby improving the heat treatment strengthening effect and enhancing the strength of the alloy. However, excessive pre-strain will inhibit the heat treatment strengthening effect, limiting the strength improvement. The results obtained in Section 4.1.1 and Section 4.1.2 just confirm this conclusion.

### 4.2. Model Fitting Results

#### 4.2.1. Uniaxial Tensile Neural Network Constitutive Model

This section takes pre-strain, heat treatment temperature, heat treatment time, and strain as inputs, and stress as the output. Therefore, we can obtain m = 4, n = 1. The learning rate of the model is set to $1\times 10^{-3}$, the target training accuracy is set to $1\times 10^{-3}$, and the maximum training iterations are set to 500,000. In this section, a single hidden layer structure is used, and through parameter tuning, the optimal number of neurons in the hidden layer is found to be δ = 5.

**Figure 14:**
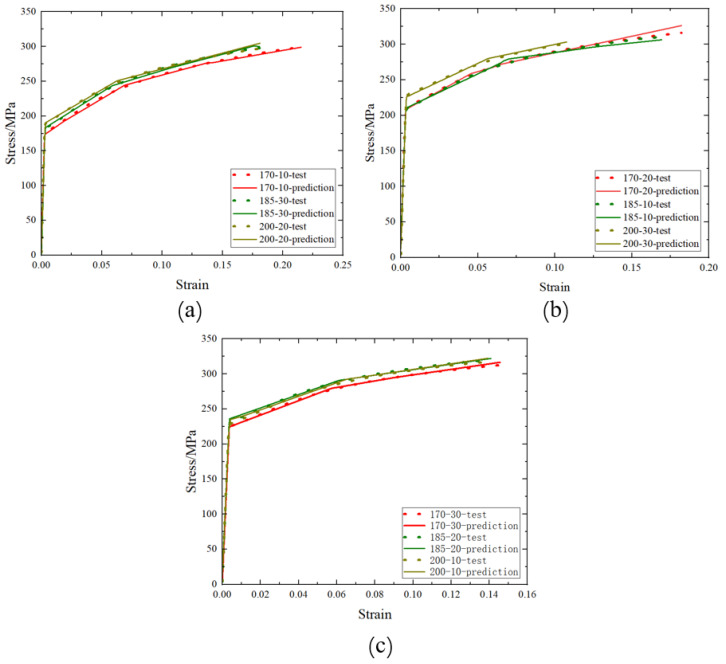
Comparison of tested and predicted values of stress–strain curves after pre-strain and heat treatment: (**a**) 6% pre-strain, (**b**) 12% pre-strain, (**c**) 18% pre-strain.

The comparison between the stress–strain data obtained from the model fitting and the experimental data is shown in Figure 14. The R^2^ for model fitting is 0.96. From the results in the figures, it can be observed that predictions closely match the experimental results, indicating that the model can accurately represent the constitutive relationship of AA6016 within a pre-strain range of 6% to 18%, heat treatment temperatures of 170–200 °C, and heat treatment times of 10–30 min. This suggests that the BP neural network model can be employed to accurately describe the mechanical behavior of AA6016 after pre-strain and heat treatment.

#### 4.2.2. Force–Displacement Curve Prediction Model

**Figure 15:**
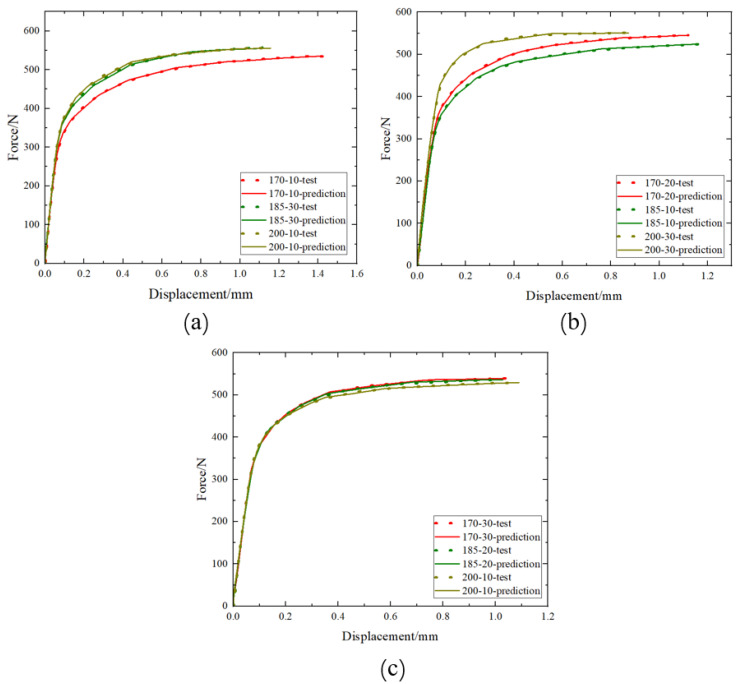
Comparison of tested and predicted values of force–displacement curves after pre-strain and heat treatment: (**a**) 6% pre-strain, (**b**) 12% pre-strain, (**c**) 18% pre-strain.

This section takes the force–displacement obtained from pure shear experiments as an example, with strain, heat treatment temperature, heat treatment time, and displacement as inputs, and force as the output. Therefore, we have m = 4 and n = 1. The model’s learning rate is set to $1\times 10^{-3}$, the target training accuracy is set to $1\times 10^{-6}$, and the maximum training iterations are set to 650. The R^2^ for model fitting is 0.99. The model’s predicted load–displacement curve matches the experimental load–displacement curve, as shown in Figure 15.

#### 4.2.3. The Influence of Model Parameters on Fitting Results

**Figure 16:**
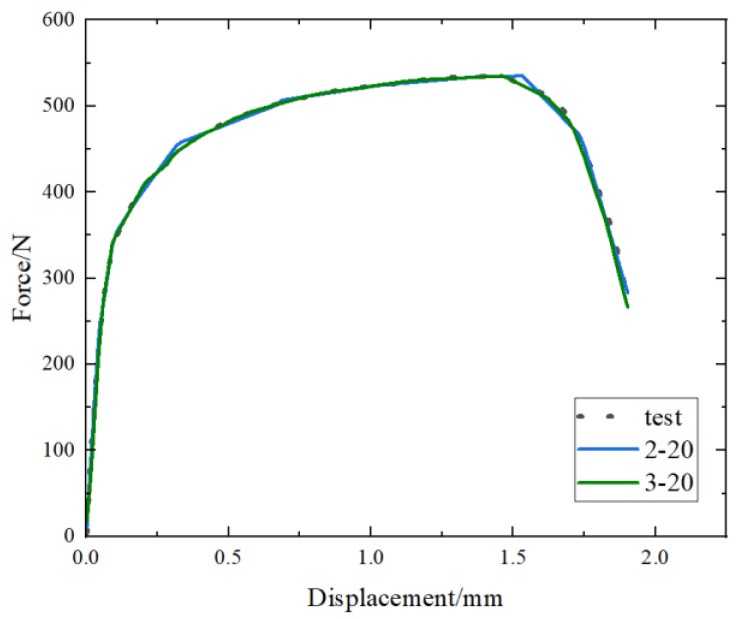
Comparison of force–displacement curves for different hidden layer parameters.

When establishing constitutive model and load–displacement curve prediction models using artificial neural network, it was found that R^2^ is very high in some cases, but the high level of dispersion of individual data prediction points results in a significant difference between the predicted curve and the experimental curve, as shown in Figure 16 (“2-20” represents two hidden layers with 20 neuron nodes, and so on in the following text). The R^2^ and RMSE of “2-20” are 0.9973 and 5.2004, while the R^2^ and RMSE of “3-20” are 0.9986 and 3.7765. It can be seen that the R^2^ of the two cases is similar, but RMSE of 3-20 is lower and the curve prediction is better. This suggests that RMSE is an important indicator in addition to R^2^.

**Figure 17:**
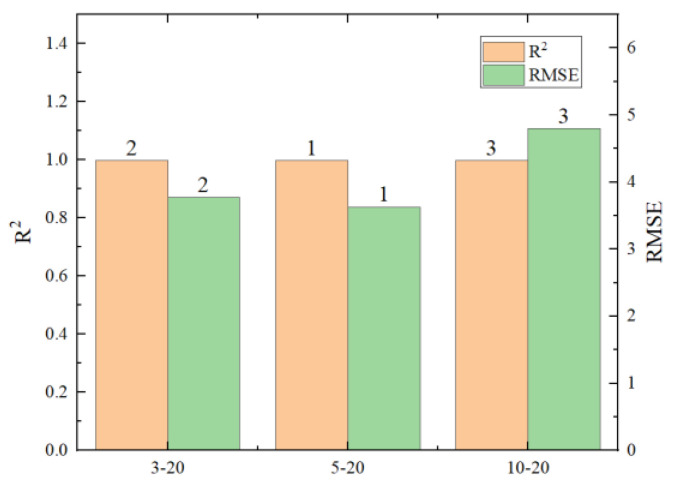
Comparison chart of different hidden layers.

**Figure 18:**
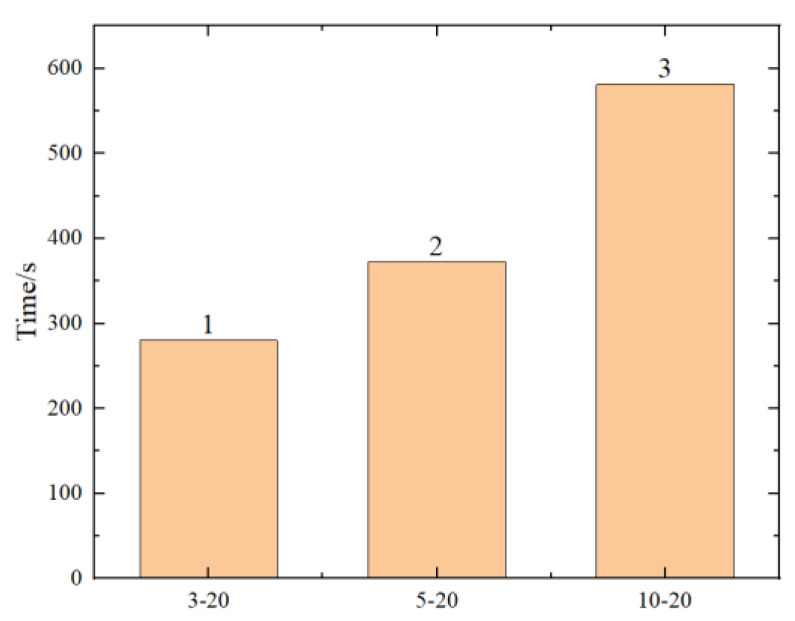
Comparison chart of prediction times for different hidden layers.

**Figure 19:**
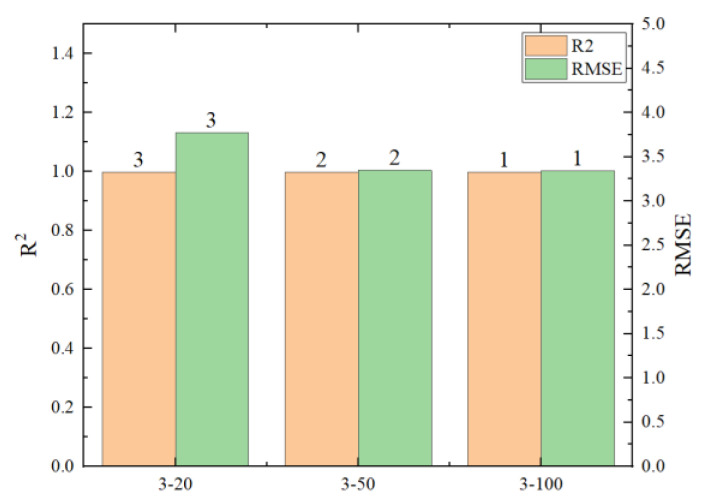
Comparison chart of different neuron nodes.

**Figure 20:**
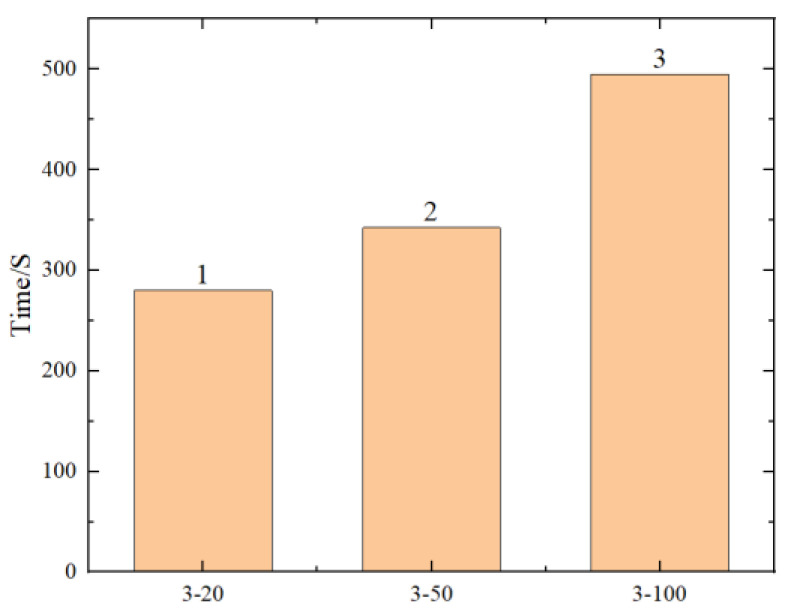
Comparison chart of prediction times for different neuron nodes.

To explore the limits of the number of hidden layers and neuron nodes in neural networks, six different scenarios were designed, as shown in Table 5. R^2^, RMSE, and the running time of the predicted results are shown in Figure 17, Figure 18, Figure 19 and Figure 20 (where the numbers on the bar charts represent the ranking). Taking “3-20” on the x-axis in the figure as an example, “3” represents the number of hidden layers and “20” represents the number of neuron nodes. From the figures, it can be observed that when the number of neuron nodes is constant, increasing the number of hidden layers, as well as increasing the number of neuron nodes when the number of hidden layers is constant, does not significantly affect the accuracy of the prediction. The changes in R^2^ and RMSE are not substantial, but the prediction time of the model increases by approximately 1-fold. Evidently, an excessively high number of hidden layers and neuron nodes is not suitable.

#### 4.2.4. The Influence of Model Architecture on Prediction Results

In a previous section, the parameters of the proposed model were determined through trial and error. The model architecture and hyperparameters could be optimized by a genetic algorithm (GA) [23]. In this section, the model architecture will be optimized by a genetic algorithm, and prediction data of the two models will be compared to study the generalization ability of the model.

Firstly, the dataset needs to be divided into a training set, testing set, and prediction set. The training set comprises six force–displacement curves at pre-strain levels of 6% and 12%. For the force–displacement curves with a pre-strain level of 18%, the curve at a heat treatment temperature of 170 °C forms the testing set, while the curves at temperatures of 185 °C and 200 °C are designated as the prediction set. Following this, both a standard BP neural network model and a genetically optimized BP neural network model were employed for training.

**Figure 21:**
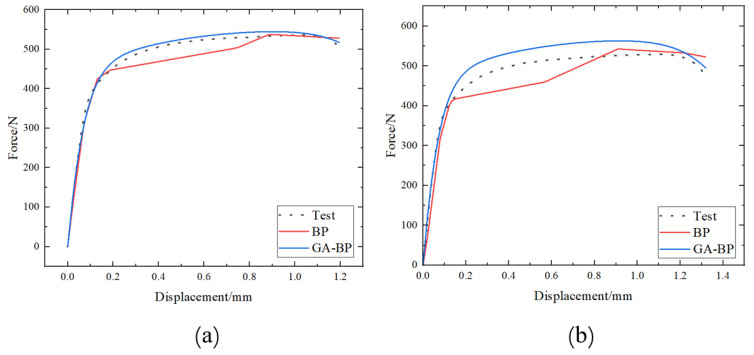
Comparison chart of prediction results between BP and GA-BP models: (**a**) 18%-185 °C-20 min; (**b**) 18%-200 °C-10 min.

The training results of the two models are shown in Figure 21, and the prediction accuracies of the two models are shown in Table 6. From Figure 21, it can be seen that the predicted values of the GA-BP model for the two curves under different process conditions are closer to the experimental values. From Table 6, it can be seen that for the curves under both of the two process conditions, the R^2^ values of GA-BP model prediction are higher than those of BP model, and the RMSE values of GA-BP model prediction are lower than those of BP model. To sum up, the BP neural network model optimized by genetic algorithm has better prediction ability.

## 5. Conclusions

Uniaxial tensile tests and fracture tests were conducted at different levels of strain before and after heat treatment. The range of strain variation was 6% to 18%, and the heat treatment temperature ranged from 170 °C to 200 °C, with heat treatment times ranging from 10 min to 30 min. Through the use of a BP neural network, an artificial neural network constitutive model and a load–displacement curve prediction model were established, leading to the following conclusions:

(1) The impact of pre-strain and heat treatment on the mechanical properties of AA6016 is primarily reflected in the increase in yield strength and tensile strength, as well as a decrease in elongation. From the analysis of variance and range analysis results, it can be observed that pre-strain has the greatest impact on the mechanical properties.
(2) The impact of pre-strain and heat treatment on the fracture properties of AA6016 is mainly reflected in the increase in shear strength and the decrease in failure displacement. From the results of variance analysis and range analysis, it appears that pre-strain predominantly influences the fracture properties of AA6016.
(3) The introduction of an appropriate pre-strain can increase the precipitation rate and quantity of the β” strengthening phase, thereby promoting heat treatment strengthening, leading to an increase in the strength of the alloy. Excessive pre-strain can suppress the precipitation of strengthening phases, thereby suppressing the strengthening effect of heat treatment and limiting the improvement in the strength of the alloy.
(4) The BP neural network prediction model can effectively fit the influence of pre-strain, heat treatment temperature, and heat treatment time on the mechanical properties and fracture performance. Through model parameter analysis, it is known that the fitting accuracy does not increase with the increase in the number of hidden layers and neuron nodes, and excessive hidden layers and neuron nodes can reduce the machine learning efficiency. The BP neural network model optimized by genetic algorithm has better prediction accuracy.

## Figures and Tables

**Table 1:** AA6016 composition table.

Composition	Si	Mg	Fe	Cu	Mn	Cr	Zn	Ti	Al
Wt (%)	0.95	0.39	0.24	0.15	0.06	0.04	0.02	0.05	98.1

**Table 2:** Table of orthogonal experiments.

Serial Number	Heat Treatment Time (min)	Heat Treatment Temperature (°C)	Pre-Strain Level (%)
1	10	170	6
2	10	185	12
3	10	200	18
4	20	170	12
5	20	185	18
6	20	200	6
7	30	170	18
8	30	185	6
9	30	200	12

**Table 3:** Mechanical properties of AA6016 before pre-strain and heat treatment.

Yield Strength (MPa)	Tensile Strength (MPa)	Elongation (%)
108 ± 2	231 ± 3	32 ± 1

**Table 4:** Mechanical properties of AA6016 after pre-strain and heat treatment.

Pre-Strain (%)	Heat Treatment Temperature (°C)	Heat Treatment Time (min)	Yield Strength (MPa)	Tensile Strength (MPa)	Elongation (%)
6	170	10	177.6 ± 2	242.3 ± 1	25.78 ± 1
6	185	20	183.2 ± 1	249.1 ± 2	22.09 ± 1
6	200	30	190.8 ± 2	248.3 ± 1	22.57 ± 1
12	170	10	212.4 ± 3	264.7 ± 2	22.79 ± 1
12	185	20	209.9 ± 2	263.5 ± 4	19.95 ± 1
12	200	30	229.1 ± 1	270.3 ± 2	15.67 ± 1
18	170	10	225.1 ± 1	271 ± 3	17.43 ± 1
18	185	20	232.4 ± 2	279.1 ± 2	16 ± 1
18	200	30	228.9 ± 1	276.7 ± 2	16.54 ± 1

**Table 5:** Hidden layer parameter table.

Number of Hidden Layers	Number of Neurons in a Layer
3	20
3	50
3	100
5	20
10	20

**Table 6:** Comparison of prediction accuracy of BP and GA-BP models.

No.	Pre-Strain (%)	Heat Treatment Temperature (°C)	Heat Treatment Time (min)	Model	R^2^	RMSE
1	18	185	20	GA-BP	0.98	10.27
				BP	0.92	23.23
2	18	200	10	GA-BP	0.85	30.37
				BP	0.76	38.32

## Data Availability

Data are contained within the article.
